# Integrated care in New Zealand

**DOI:** 10.5334/ijic.678

**Published:** 2011-11-18

**Authors:** Jacqueline Cumming

**Affiliations:** Health Services Research Centre, School of Government, Victoria University of Wellington, PO Box 600, Wellington, 6035, New Zealand

**Keywords:** integrated care, health policy, health reforms, New Zealand

## Abstract

**Background:**

New Zealand’s health system has long been seen as providing highly fragmented, poorly co-ordinated services to service users. A continuing policy challenge has been how to reduce such fragmentation and achieve more ‘integrated’ care, that is, ‘co-ordinated’ care that provides a ‘smooth and continuous’ transition between services, and a ‘seamless’ journey as service users receive health, support, and social welfare services from a range of health and other professionals.

**Description of policy practice:**

The paper takes as its starting point the view that achieving integrated care needs to be supported by a “coherent set of methods and models on the funding, administrative, organisational, service delivery and clinical levels” [1]. The paper considers how fragmentation in financing, planning, funding, and service delivery have contributed to poorly co-ordinated care in New Zealand; discusses how integrated care was to be supported by recent major reforms to the health system and whether such reforms have succeeded or not in achieving more integrated care for service users; and discusses the challenges New Zealand still faces in achieving more integrated care over the next few years.

**Discussion and conclusion:**

The paper concludes that although key financing, planning, funding and service delivery reforms aimed at delivering more integrated care to service users have succeeded in integrating planning and funding functions, few changes have occurred in the ways in which services are provided to users. It is only now that significant attention is being paid to changing how services are actually delivered in order to achieve more integrated care, but even then, change appears to be slow, and significant challenges to integrating care in New Zealand remain to be resolved.

## Introduction

New Zealand’s health system has, for many years, been seen to provide highly fragmented, poorly co-ordinated services to service users. Fragmentation arises because service users receive care from a wide range of professionals working in a large number of provider organisations, while a lack of information sharing and liaison between these professionals and provider organisations is seen to result in poorly co-ordinated care.

A continuing policy goal in New Zealand has been to reduce such fragmentation and achieve more ‘integrated’ care. This paper provides an overview of recent policies to better integrate care in New Zealand. It considers how fragmentation in financing, planning, funding, and service delivery have contributed to poorly co-ordinated care in New Zealand; discusses how integrated care was to be supported by recent major reforms to the health system and whether such reforms have succeeded or not in achieving more integrated care for service users; and discusses the challenges New Zealand still faces in achieving more integrated care over the next few years.

### Definitions

The concept of ‘integrated care’ has not always been well defined in New Zealand. In its most narrow form, integrated care is seen as an important outcome for service users, where the care they receive is ‘co-ordinated’ [2–5]. More often than not, it also includes ensuring good access to primary care providers, who should co-ordinate care [2, 4, 6]. ‘Integrated care’ has also at times referred to the linking together of key planning, funding, and service delivery activities to support co-ordination [2, 4], and a single budget for integrated service delivery organisations which would provide a wide range of services to their enrolled populations [2, 7, 8].

For the purposes of this paper, integrated care is service delivery that provides a ‘smooth and continuous’ transition between services [3], i.e. ‘co-ordinated’ care [2–5], with co-operation and collaboration across services [2, 7] and a ‘seamless’ [9] journey for service users, as they receive health, support and social welfare services from a range of health and other professionals. Much attention in New Zealand has been focused on integrated care within primary care services (‘horizontal’ integration); and between primary and secondary care services (‘vertical’ integration); public health and curative services; health and support services (e.g. personal support services for people with disabilities or for older people); and health and social welfare services (e.g. welfare, housing, and employment services) (‘inter-sectoral’ integration).

The paper takes as its starting point the view that achieving integrated care needs to be supported by a “coherent set of methods and models on the funding, administrative, organisational, service delivery and clinical levels designed to create connectivity, alignment and collaboration within and between sectors’ [1]. Thus, the paper considers how key functions in the New Zealand health sector—discussed here as financing, planning, funding, purchasing, and service delivery functions—have been reformed in recent years to support or achieve more integrated care. In the New Zealand context, financing refers to the ways in which health services are paid for (e.g. taxes, user fees); planning refers to needs assessment and priority setting activities; funding refers to a passive approach to paying providers for their services; and purchasing is a more active approach to allocating resources, including tendering for services and the use of formal contracts to manage service delivery and performance.

### Background

New Zealand’s problems with fragmentation of health service delivery go back to the mid-to-late 1800s, when early governments supported a mix of central and local government, voluntary, and private financing; and a mix of public, private for-profit, and private not-for-profit provision by many independent providers and provider organisations, to ensure the delivery of services to the growing New Zealand population [3, 4].

The first major attempt to reform these arrangements came with the introduction of the Social Security Act in 1938 [4], which aimed to introduce universal free care for many health services, as a part of plans to establish a single, national health service [10, 11]. Free public hospital and maternity care were successfully introduced from the 1940s onwards, but the government could only reach agreement with the medical profession to partially finance general practitioner services, leaving service users to pay fees to access such care [4], a situation which continues to this day.

Meanwhile, there were major separations in the planning, funding, and provision of services; with,

- Public health services the responsibility of a central Department of Health, with services provided through 18 district offices [12];
- Primary care services funded through a separate division of the Department of Health, with general practice services delivered by community-based, privately-owned, small general practices led by general practitioners, who acted as ‘gatekeepers’ to a range of referred primary and secondary care services, and with separate subsidies funding diagnostic tests delivered by private laboratories, pharmaceuticals delivered by private pharmaceutical companies, and pharmaceutical dispensing services delivered by private pharmacists;
- Local publicly-owned hospitals providing specialist in-patient and out-patient and some community-based services (such as district nursing services). Secondary mental health care planning and funding roles were not integrated with other hospital service planning and funding roles until the 1940s, while mental health service delivery was not devolved from the central Department of Health to local hospitals until 1972 [4];
- Other community-based services delivered by a range of not-for-profit community organisations, such as Plunket, which provides well child services;
- Services for people with disabilities (including those with physical, age-related, intellectual and psychiatric disabilities) also fragmented, with the health sector funding and providing hospital care, and the social welfare sector funding and providing community services, with many services delivered by community-led, not-for-profit organisations (such as the IHC for children with intellectual disabilities and CCS for children with physical disabilities) [13].

These arrangements have long been seen as problematic with respect to achieving integrated care in New Zealand. First, the partial financing of general practice services and on-going increases in service user fees established an important barrier to access to such services [4, 14, 15]; made it difficult to fully link general practice planning and service delivery with other service planning and delivery; and made it difficult for primary care service providers to take the lead in co-ordinating care. Second, the separation of roles resulted in insufficient co-ordination in planning and delivering services, leading to duplication of, and gaps in, service availability, while the use of different criteria to access services made it difficult for service users to consistently get the care they needed to improve their health. Third, a lack of information sharing and liaison between providers were seen to result in poorly co-ordinated care: service users could slip through gaps in the system or be seen by multiple providers for the same condition, information might not be shared or could go missing, tests might be duplicated, harm could occur from the use of incompatible medications, and service users might receive different health advice from different health providers [3, 4, 6, 16, 17]. The overall impacts of these arrangements were seen to be poor quality of care and a waste of scarce health resources.

Prior to the 1980s, New Zealand policy makers attempted many times to reform New Zealand’s health care system, and more often than not more integrated care has been an important goal of such reforms. A number of early policy documents refer to the desirability of creating a ‘single, national health service’ [10, 11], while various strategies proposed major structural reforms, in particular to planning, funding, and service delivery arrangements, with a view to, *inter alia*, delivering more integrated care [3, 4, 11]. Various political difficulties stymied numerous early attempts at reform [3, 4, 18–20], however, and it was not until the 1980s that key reforms began to successfully integrate key functions and provide a more conducive environment for achieving integrated care.

## The first steps: integrating planning and funding for public health and hospital services—Area Health Boards in the 1980s

The first major set of recent reforms, in the 1980s, focused on integrating planning and funding functions, and public health and secondary care service provision, at a district level. Fourteen geographically-based Area Health Boards were established, each responsible for planning all health services, including primary care services, in their district. The aims of these reforms were to encourage a focus on the health of a (geographically-) defined population, streamline and co-ordinate planning between sectors, and increase the health system’s focus on health protection and disease prevention [17, 21]. The population health focus established by these reforms remains today, but as the funding and provision of primary care services remained separate, Area Health Boards were never likely to succeed in developing the primary care role further, stymieing any attempts at improved integration of primary and secondary care service delivery. Unfortunately, Area Health Boards were not in place very long to show the benefits of more integrated planning and service delivery before the structure of the health sector was once again reformed.

## Full integration of planning and funding, competition, and integrated primary care providers—The ‘Purchaser-Provider Split’ in the 1990s

The 1990s reforms were aimed at improving access to services and the overall efficiency of the New Zealand health system, including through the provision of more integrated care and an increased emphasis on primary care [2]. The reforms further integrated planning and funding responsibilities, this time into the hands of four Regional Health Authorities. Thus, the funding for public health^1^, primary, secondary, other community services, and the previously social welfare-organised disability support services for people with intellectual and physical disabilities, was all integrated, so that the Regional Health Authorities could more consistently fund services and encourage collaboration, shift resources between previously separated budgets to support the most cost-effective services and providers, and encourage a greater emphasis on prevention and primary care, which in turn would enable increased attention to be paid to more co-ordinated care [2].

These same reforms separated planning/funding/purchasing from provision roles (with the former placed in the hands of the four Regional Health Authorities), established formal contracting mechanisms throughout the New Zealand health system, and promoted competition in the delivery of health services. These arrangements had both negative and positive effects on incentives to integrate care. On the one hand, they enabled the new purchasing authorities to allocate resources to new primary and community care providers, enabling more choice and improved services, particularly for Māori [15, 23] and Pacific [24] populations, where mainstream services had been seen to be unresponsive to meeting these groups’ needs [6]. This coincided with on-going de-institutionalisation in the care of the elderly and mental health services, with private for-profit and not-for-profit organisations taking over service delivery and many services moving out of hospitals and into community settings [13]. This proliferation of providers arguably created more fragmentation, while the competitive elements of the reforms reduced incentives for service providers to work together to improve health [20]. Further fragmentation developed as independent midwives gradually took over the delivery of maternity services from general practitioners [25].

On the other hand, the 1990s reforms also resulted in the development of new primary care provider networks [26]. Such organisations would eventually go on to promote more integrated care in New Zealand. These general practitioner-led networks (the most common being Independent Practitioner Associations [27]) provided business and support functions to general practices, developed new primary care planning and analysis functions, focused on improving quality of care, increased community involvement in primary care planning, and provided a range of new services across general practices [27, 28]. A number also developed new funding arrangements [29], including capitation, risk-related budgets, and in one case, a global budget, which provided greater incentives to manage costs and allowed more flexibility in the allocation of resources to different services [30].

The late 1990s saw the amalgamation of the four Regional Health Authorities into a single, national Health Funding Authority. The Health Funding Authority identified service integration as a key development strategy, along with an emphasis on primary care, and developed two strategies to promote improved integration of services. From this point on, New Zealand had a real policy focus on trying to achieve more integrated care.

First, the Health Funding Authority called for tenders from providers to develop integrated care initiatives as national pilot projects [8]. An integrated system would rely on: collaboration across services; a focus on health promotion and prevention of disease, avoidable complications, and disability; consumer support services for people managing their own health; effective information and management systems; a focus on evidence-based practice; partnerships between service users and professionals; and achieving improved health and cost-effective service delivery [8]. The Health Funding Authority sought pilot projects that could test the impact of eight hypotheses on health outcomes and cost-effectiveness, with projects that would involve the use of decision-making guidelines; contracting strategies that aligned incentives and promoted collaboration across traditional service boundaries; integrated funding; budget responsibility for a wide range of primary care services, for a specified bundle of services (e.g. mental health, asthma, diabetes), or for a full range of services; integrated service networks by Māori for Māori; and consumer choice [8].

Thus, some projects would encourage more local responsibility for budgets and service planning through devolution of funding from the Health Funding Authority to local purchasing organisations (such as Independent Practitioner Associations). This type of project never occurred [20, 31], however, as it was felt that a series of risks attached to devolving funding were not adequately mitigated in the submitted proposals [20].

Other projects were more focused on changing service delivery, and nine such integrated care pilot projects were eventually funded by the Health Funding Authority. These ‘demonstration projects’ varied significantly in terms of size, scope and intention, and covered a range of services, including child health, diabetes management, family/whānau support, elder care, heart failure, paediatric asthma, and mental health services. An evaluation of the pilots found evidence of improved co-operation between the providers engaged in the pilots, and the use of a wide range of integration tools (such as clinical pathways and guidelines, improved information systems, shared care, etc.), but there were little data to show whether the pilots improved integration from the service user perspective, health outcomes improved, or new service delivery arrangements would be cost-effective [31, 32].

Second, the Health Funding Authority set out a plan that would see the development of general practice services as multi-disciplinary teams, including allied health workers, serving populations of at least 30,000 people [7]. Such Primary Health Service Organisations would focus on improving the health of their enrolled populations [33], increasing the delivery of services in primary care settings, and managing patient care across primary and secondary care services, as well as managing financial risk [20]. Effectively, Primary Health Service Organisations would become local managed care primary care organisations [34]. Such arrangements were never able to be introduced, however, before the next restructuring.

## Local level initiatives—District Health Boards in the 2000s

In 2000, the New Zealand Public Health and Disability Act established 21 (now 20) District Health Boards, re-integrating funding and provision of hospital services, with District Health Boards also responsible for planning and contracting for community services, and later, primary care. Some ‘dis-integration’ of planning and funding also occurred with this model: some services (e.g. well-child, telephone helpline, mobile surgical, and sexual health services) became the responsibility of the Ministry of Health (often to allow single national provider organisations to provide services under one contract rather than 20), as did public health services (to protect public health funding), and disability support services for those aged 65 and under (as a result of concerns over the potential for further ‘medicalisation’ of disability if District Health Boards became responsible for such services [35]). Funding for primary maternity care (largely now delivered by independent midwives) was also never devolved to District Health Boards.

Achieving more integrated care continued to be a focus for District Health Boards during the 2000s, in particular for people with chronic illnesses. Much activity occurred at a local level, although very little published material is available from this time on changes to service delivery. Counties Manukau District Health Board has, however, published material on the many projects they undertook to improve service delivery during the 2000s. The projects initially developed because of concerns about poor co-ordination within primary care and between primary and secondary services, and the need to reduce pressure on hospital services. A range of initiatives was implemented: a number focusing on identifying high users of hospital services, improving their links with primary care services, or increasing the role of primary care providers in care delivery; others using improved information systems to reduce duplication and prevent gaps in service delivery; and others focusing on improving discharge planning, increasing the use of treatment and referral guidelines, and developing care co-ordination tools to improve care. Evaluations found some important achievements and improvements in health, including statistically significant improvements in diabetes care outcomes, and reductions in blood pressure and cholesterol, but reductions in smoking rates and increases in the use of ACE inhibitors, beta blockers, statins, and aspirin were not statistically significant [36, 37].

## The Primary Health Care Strategy and formalisation of integrated primary care providers—the 2000s

In 2001, the Primary Health Care Strategy was released [38]. The Strategy has a focus on improving population health, the removal of health inequalities, and improving the co-ordination of care [38]. Significant new funding was provided to reduce the costs of primary care services, with a view to enhancing the role of primary care in New Zealand. Improved co-ordination of services was to include a collaborative, multi-disciplinary approach by health professionals across and between all levels of the health sector, as well as inter-sectoral work (with a range of social welfare agencies) to address health issues [38]. The Strategy took the idea of having meso-level organisations in primary care, such as Independent Practitioner Associations and the earlier planned Primary Health Service Organisations, further; with new Primary Health Organisations to be established nation-wide, held responsible for the health of their enrolled populations, and funded on a capitation basis [38]. Independent Practitioner Associations and the community-, Māori- and Pacific-led providers established during the 1980s and 1990s have played major roles within these Primary Health Organisations.

Evaluations of the Primary Health Care Strategy have identified significant gains, including the establishment during the 2000s of around 80 new Primary Health Organisations to lead primary care service development and integration, most of the population being formally enrolled with Primary Health Organisations, reduced user fees, increased service provision, and increased consultation rates [39–46]. There have also been improvements in performance amongst Primary Health Organisations in achieving key targets (e.g. in breast and cervical cancer screening rates, and flu and child vaccination rates, including for high needs population groups [47, 48]).

## Alliances and integrated family health centres and clusters—the 2010s

In spite of all the changes discussed above, by the end of the 2000s, there remained concerns that very little had changed in terms of how services (especially primary care services) were actually delivered to service users [9, 45, 49, 50]. In particular, it has been argued that New Zealand has not worked hard enough to improve co-ordination of care [41, 45] and this is also seen as a symptom of the failure to identify what a ‘comprehensive’ model of primary care might look like in New Zealand and how it might be delivered here [45].

The focus now is on “New models of care which see the patient rather than the institution as the centre of service delivery and which aim to promote a more seamless patient journey across community, primary, and hospital sectors, greater use of primary and community care, and the shifting of care ‘closer to home’” [49, 51]. To facilitate these changes, the Government, in September 2009, released a request for expressions of interest to deliver new models of care [52]. More than 70 responses were received, with nine groups (now called ‘Alliances’) selected to subsequently proceed to implementation [53] (see below). At the same time, the government is seeking reductions in the number of Primary Health Organisations to improve the infrastructure for, and reduce the costs of, primary care service planning in New Zealand [53, 54].

These reforms are leading to changes once again in the structure of the health sector.

First, there has been a reduction in the number of Primary Health Organisations in New Zealand, from around 80 in 2010 to 36 in July 2011 [55].

Second, the Alliances are developing new collaborations to plan and deliver services. There are new regional macro-level networks in Auckland [56] and Canterbury [57], involving a wide range of organisations in planning, funding, and delivering services. There are new meso-level networks of Primary Health Organisations, with four Primary Health Organisations working together in the Midlands region [58], and a National Māori Primary Health Organisation Coalition bringing together 11 Māori-led Primary Health Organisations [59]. There have also been amalgamations of Primary Health Organisations in four districts, resulting in a new Pacific-led Primary Health Organisation to better co-ordinate services and build critical mass for the Pacific sector in Auckland [58], and, in the other three districts, a single Primary Health Organisation now plans and funds all primary care services in each of their respective districts [58, 60, 61].

Third, each Alliance is planning to implement particular initiatives to improve co-ordination of care, through devolution of funding and services from District Health Boards into the community; increased co-ordination of services between primary care providers and hospitals; the development of integrated family health care centres (multi-practitioner centres), co-located clinics, and ‘clusters’ of providers to deliver more integrated services; more nurse-led services and multi-disciplinary teams; improved co-ordination across Pacific primary care; and devolution of services to Māori communities and the development of whānauora^2^ (‘family well-being’) models of care to improve Māori health [53, 58]. The ‘Alliances’ are must develop a single governance group and integrated operational management structure [64] and use ‘alliance’ contracting mechanisms to advance their proposals; such contracts are generally set up such that all information (including financial information) is disclosed, objectives are shared, and rewards are distributed based on actual outcomes.

Finally, the government has also introduced a new set of policies and initiatives (confusingly also called ‘whānauora’) to enhance co-ordination between the health and social services sectors, including community and social development, Māori development, health, education, justice, and housing, for high needs whānau. These initiatives focus on the development of single whānauora contracts, and are aimed at enabling Māori providers from the various sectors to work together so that a coherent approach to whānau development can occur [65].

## Discussion

For New Zealand, achieving integrated care has long been a key policy challenge, and many of New Zealand’s major structural reforms to the health system have included improved integration as a key goal of reform.

Although more integrated care has long been a policy goal in New Zealand, we in fact know very little about how New Zealand service users view and experience their health services in relation to fragmentation and integration. What research is available does show New Zealanders reporting problems with poor communication between services, especially between primary and secondary care services [66, 67]. A 2010 Commonwealth Fund survey found that New Zealand (along with the USA) had the highest proportion (69%) of respondents agreeing that a regular doctor always or often co-ordinates care. However, those with two or more chronic conditions report more problems in New Zealand (26% vs. 19% for those with no chronic conditions) and there are reported problems with receiving conflicting information from different health professionals (with 18% reporting this occurred); the specialist not having the reason for visit/tests from the service user’s regular doctor (22%); the regular doctor not being up-to-date about specialist care (30%) or being informed or up-to-date about care received in the emergency department (22%); failure to communicate test results (21%); and perceptions of inefficient or wasteful care where care was seen to be poorly organised or co-ordinated (with 12% reporting this) [66]. These results suggest that New Zealand service users do have problems with fragmented care, with around 20% reporting problems.

Many New Zealand reforms have focused on integrating key functions around financing, planning and funding and providing more integrated care for service users. In making sense of New Zealand’s recent reforms and their impact on achieving more integrated care, Table 1 summarises New Zealand’s key reforms within a framework that draws on recent work by Ling et al. [68], Fulop et al. [69], and Lewis et al. [70].

The columns in the Table set out the years and levels at which reforms have taken place:

- the *micro* level—activities that promote integration among individual practitioners working in a single organisation (e.g. doctors and nurses working in a single general practice);
- the *meso* level—activities that promote working between organisations (e.g. general practitioners and specialists); and
- the *macro* level—activities that promote organisation-to-organisation collaboration, such as policy agreements or contractual arrangements, financial arrangements, such as pooled budgets or joint budget holding, employment of staff in a single organisation; or the establishment of new organisations that oversee these tasks.

The Table rows set out what is being integrated: high-level planning and funding functions; service budgets; service planning and support functions; and service delivery functions (including for single conditions and for specific populations).

As can be seen from the Table, many New Zealand reforms have occurred at the macro level, with an emphasis on integrating planning and funding for health services. It is perhaps not surprising that so much activity has occurred at the macro level in New Zealand, given central government financing of key services, coupled with central government ownership of key organisations, such as Area Health Boards, Regional Health Authorities, the Health Funding Authority, and District Health Boards.

Unfortunately, New Zealand’s experiences with macro-level reforms also show that such reforms, on their own, are insufficient to actually deliver more integrated care—each reform having generally failed to more clearly link meso-level and micro-level reforms together to achieve more integrated care [71].

A key reason for this lies in the separate and private financing and provision of primary care services in New Zealand, where primary care has always been seen as a key component of integrated care in New Zealand, but where long-standing distrust between New Zealand governments and primary care providers continues to dictate the policy choices governments can and do make in New Zealand health care [10, 11, 72].

On the surface, recent reforms do appear to have improved the position of primary care in New Zealand and increased our opportunities for achieving integrated care. The unexpected development of primary care networks (such as Independent Practitioner Associations) in the 1990s, followed by the introduction of Primary Health Organisations in the 2000s, have allowed meso-level organisations to begin to play a role in strengthening primary care services and promoting more integrated care. The re-introduction of universal financing and increased levels of financing for primary care services during the 2000s also assisted in focusing attention on the roles that an enhanced primary care service might play in better supporting integrated care. The full move to capitation funding in primary care in the 2000s—taking the focus away from general practitioner primary care service delivery and potentially enabling a wider range of providers to deliver primary care services—has also been seen as an important precursor to achieving more integrated care.

However, concerns still remain that insufficient change in actual service delivery has been achieved in recent years. There appear to be a number of reasons for this. First, insufficient attention was paid to identifying new models of service delivery during the implementation of the Primary Health Care Strategy during the 2000s in particular, with too much policy attention focused on funding and new infrastructure [45], where New Zealand policy makers have had more direct control. Second, issues relating to the lack of clarity around the roles of Primary Health Organisations [45, 46]; a lack of positive engagement between government and general practice [45, 73]; and little attention being paid to leadership, management and organisational development [45], were all factors that played a role in limiting service delivery change in New Zealand primary health care during the 2000s. Third, budgets for a wide range of primary care services remain outside of Primary Health Organisation control, including pharmaceutical dispensing and diagnostic services, and midwifery services.

Since the release of the Better, Sooner, More Convenient policy document and the election of a new government in 2008, significant attention is now being paid nationally to actually changing service delivery arrangements with a view to achieving more integrated care. But, as the Table shows, even these changes are also accompanied by reforms at the meso and macro levels. Thus, Primary Health Organisations are being encouraged to amalgamate to improve their capacity and capability to manage change, in the face of concerns that not all such organisations have performed well [34, 46]. In addition, two new macro-level Alliances are being developed. These seem to reflect a need for a less hierarchical arrangement to be developed within the system, given concerns that District Health Boards both provide services and contract for primary care provision and hence may not always have an interest in promoting the greater delivery of primary care services in the community, and the fact that that existing primary care organisations largely represent general practice services only, with many other primary care and community providers still outside such arrangements.

Is there any evidence that the new arrangements are making a difference and leading to more integrated care? A feature of recent reforms has been the failure to document, evaluate and share innovations and lessons learned in trying to effect change in service delivery [45] and unfortunately, a continued lack of research means we know very little about what is happening now to better integrate care. Anecdotal evidence, however, suggests that change is slow and patchy, and occurring often at a very local level. In terms of a continuum along which integrated care organisations might be achieved, ranging from full segregation, to linkage, co-ordination, co-operation and full integration [74, 75], it also appears that many New Zealand reforms are at only the beginning stages of integration, with attention being paid to improved information sharing through electronic means, as well as to co-locating at least some services in the form of integrated family health centres. It is not always clear how far integrated family health centres arrangements go beyond co-location to develop greater co-ordination and co-operation, while full integration seems as far away as ever.

Thus, in spite of many recent reforms, New Zealand still faces significant challenges in achieving more integrated care.

The main challenge, at the micro level, lies in encouraging a wide range of providers who currently operate separately at the primary care level—general practitioners, primary care nurses, pharmacists, midwives, social workers, physiotherapists, occupational health therapists, community workers, district health nurses, and public health nurses—to increasingly work together, perhaps eventually under a single budget to promote a more co-ordinated approach. We also need primary care services to better link with secondary care and support services, including with fiercely independent not-for-profit organisations. It remains to be seen how well all these providers collaborate, but it is clear that change is very slow, no doubt due to concerns over leadership and budget control.

Separate evaluations in New Zealand of various integrated care initiatives have found that similar factors are critical to success, including: a focus on changing cultures and attitudes and the need to take the time to develop co-operation and collaboration [31, 32]; developing early, formal relationship agreements with Māori and Pacific populations [31, 32]; enthusiastic leaders, champions and key participants [31, 32]; political commitment to change [31, 32]; involvement of clinical staff [36]; reassurance for providers about privacy issues when sharing information [36]; close monitoring of project progress; realistic timeframes; and adequate initial funding [36]. Important barriers to integration have included a lack of funding integration, and ‘patch’ protection and competition between providers [31, 32]. International evidence likewise notes the importance of physician–management partnerships, effective leadership and collaborative cultures [71]. Careful attention to all these factors is needed in New Zealand, as well as a balance between taking the time to develop new cultures and ways of working and ensuring that change does occur and old ways of working do not stay embedded in the system.

At the meso level, it also remains to be seen how successful each of the different forms of network New Zealand now has working in primary care—Independent Practitioner Associations, amalgamated Primary Health Organisations, Alliances—are in achieving change. Earlier evidence suggested that the performance of networks in New Zealand was patchy [34, 46]. Again international evidence suggests that clinical engagement is key to the success of such organisations, and although the size of such organisations may or may not influence their ability to effect change, there is evidence that such organisations should not become “unduly complex, bureaucratic and distant from its professional stakeholders” [34]. There is a concern that the recent amalgamations of Primary Health Organisations and the large size of New Zealand’s Independent Practitioner Associations may hamper further change if they become too remote from their members.

At the macro level, a number of important services remain the responsibility (in planning and funding terms) of the Ministry of Health. If New Zealand is serious about better integrating care, such services will eventually need to be devolved to lower levels within the system. This will continue to be contested, with a key need for all organisations to show their willingness to take on a broad approach to achieving health and for different professional groups to work together.

Finally, New Zealand continues to face a key problem in working to better integrate care: the extent of user fees that continue to form part of financing arrangements for primary care services in New Zealand. As well as providing barriers to access to primary care services, such fees continue to make it difficult for central government to manage primary care services. Future plans to increase the role of primary care organisations in managing larger budgets will face the problem that such organisations can manage financial risk simply by raising user fees rather than through more efficiently delivering services and may also profit from widening the range of services upon which fees are applied [76]. This issue needs urgent attention and an agreed way forward with primary care stakeholders [73].

### A longer term view

If integrated family health centres do develop significantly, with an extended primary care service delivered in an increasingly integrated way through information sharing, and team work, and where such centres work with meso level primary care organisations that hold significant primary care budgets and risk, New Zealand will get closer to developing (vertically) integrated primary care service delivery organisations. As such new models develop, the question about who holds the budgets for at least some secondary care services will also arise. Currently, District Health Boards are budget holders for a wide range of services, but meso-level primary care organisations may also be considered possible budget holders for secondary care services, increasing incentives to keep people out of hospital, and providing opportunities to better integrate care as users move between primary and secondary care services. However, the fledgling integrated primary care organisations New Zealand has are privately-owned and New Zealanders may not support them holding such large budgets for health care delivery, while the existence of too many primary care organisations holding budgets for secondary care would significantly increase transaction costs for hospitals and potentially seriously destabilise such services. Any such devolution of funding also requires careful thought as to how District Health Boards, with significant capital requirements, can continue to fund all their overheads, as well as enabling a fair allocation of resources to primary care providers who take new services on.

Even harder to achieve may be the fully integrated models, such as those run by Kaiser Permanente and Geisinger in the USA, which link funding as well as a wide range of primary and secondary care provision closely together, and which have been shown to perform well [71]. This would involve individual New Zealanders choosing to receive all their care from a single organisation, and a number of such organisations competing against each other to serve New Zealanders. It seems unlikely that such models could work in New Zealand, as the limited number of hospitals providing full (including emergency and intensive care) services and the need for hospital services to be delivered at a local level to a widely dispersed population means only those living in Auckland, Wellington and Christchurch would have a choice of integrated provider [76]. But choice of primary care organisation, with integrated primary care arrangements and mechanisms to co-ordinate secondary and support care, is more feasible; although, as noted above, current reforms leading to the amalgamation of Primary Health Organisations have already left both providers and service users without a choice in some parts of the country.

## Conclusions

New Zealand has long focused on attempting to deliver more integrated care across a wide range of health, support, and social welfare services. Many attempts at reform have focused on re-organising planning and funding arrangements to strengthen the role of primary care services in service delivery; improve the planning of services; and have all funding together to reduce silos, thereby encouraging the allocation of resources to cost-effective services, and providing more flexibility in service provision to meet health needs. In spite of many reforms, many budgets and services remain in silos, and actual service delivery remains fragmented. Significant policy attention is now focused on integrating service delivery, particularly within primary care, between primary care and secondary care services, and inter-sectorally. It is too early yet to say how these reforms are progressing, but the building blocks—improved primary care financing and improved access to primary care services, integrated planning and funding, a local focus through District Health Boards, and stronger primary care organisations—may make it more likely that change may be achieved this time. Even then, a sustained effort is likely to be needed to overcome the many likely challenges involved.

The most pressing need in New Zealand now is for comprehensive research and evaluations of current changes—we need to know more about the changes that are actually occurring in New Zealand and what lessons can be learned from both successes and failures. In particular, we have very little information about how service users in New Zealand think about integration and whether or not improved integration from a service user perspective is in fact being achieved. We know nothing about the range of mechanisms that are being used to integrate services, nor how successful each is at effecting change. We also know nothing about the advantages and disadvantages of the various primary care networks New Zealand now has and how each is working to effect change in service delivery. With improved information, we can also better consider the implications of current changes and reach a clearer vision of the future arrangements that might work best in New Zealand, which recognises that fully integrated delivery models may not be possible. Without such research and evaluation, we cannot be sure that the resources currently being used to support more integrated care are actually achieving the goals of more integrated care, improved health, and improved efficiency.

## Figures and Tables

**Table 1.** Levels and Types of Integration in New Zealand 1980s–2010s

Levels of integration	1980s			1990s			2000s			2010s		
	Micro	Meso	Macro	Micro	Meso	Macro	Micro	Meso	Macro	Micro	Meso	Macro
What is being integrated?												
**Planning and funding functions**			Area Health Boards—Public health, primary and secondary care			Regional Health Authorities—All health and support services Health Funding Authority—All health and support services			District Health Boards—Most services			Regional alliances—Planning/funding for some services; providers included District Health Boards—Most services Whānauora contracts—High needs populations
**Service budgets**			Area Health Boards—Public health, primary and secondary care		Some capitation Some pharmaceutical and laboratory budget contracts Global budget (for one Independent Practitioner Association)			Capitation for Primary Health Organisations (first contact services)			Capitation for Primary Health Organisations (first contact services)	
**Service Planning and Support**			Area Health Boards—Public health and secondary care		Independent Practitioner Associations			Primary Health Organisations	District Health Boards—Public health and secondary care		Primary Health Organisations (amalgamations)	District Health Boards—Public health and secondary care
**Services**			Area Health Boards—Public health and secondary care				Local initiatives	Local initiatives	District Health Boards—Public health and secondary care		Local initiatives Integrated family health centres and clusters	District Health Boards—Public health and secondary care
**Services for a single condition**				Integrated care pilots	Integrated care pilots Local initiatives		Local initiatives	Local initiatives			Local initiatives	
**Services for a specific population**				Integrated care pilots				Māori and Pacific Primary Health Organisations			Māori and Pacific Primary Health Organisations Whānauora organisations—Provision for high needs populations	

## Footnotes

1 Planning and funding roles for public health services were at first separated and allocated to a Public Health Commission; the Commission was dis-established in 1995 [22], and public health funding allocated to the four Regional Health Authorities instead.

2 ‘*Whānauora*’ is the stated goal of the government's Māori Health Strategy, He Korowai Oranga which seeks to achieve whanauora or *Māori families supported to achieve their maximum health and wellbeing* [62]. The term is now be used to refer to a philosophy (which focuses on the health of the whole whānau (family), not just the health of the individual), a distinct model of practice (embracing the health and social service sectors), and an outcome in its own right [63].

## Reviewers

**Pauline Barnett**, Associate Professor, Health Sciences Centre, University of Canterbury, Private Bag 4800, Christchurch 8140, New Zealand

**Robin Gauld**, Associate Professor of Health Policy, Department of Preventive and Social Medicine, University of Otago, New Zealand

**Nicholas Mays**, Professor of Health Policy, Department of Health Services Research and Policy, London School of Hygiene and Tropical Medicine, 15–17 Tavistock Place, London WC1H 9SH, UK

**David Perkins**, Associate Professor, Director Centre for Remote Health Research Broken Hill Department of Rural Health University of Sydney, Australia
